# Linking entrepreneurial spirit and entrepreneurial environment perception to entrepreneurial intention: Moderation by entrepreneurial role models

**DOI:** 10.1371/journal.pone.0352807

**Published:** 2026-07-06

**Authors:** Biyan Xiao, Xiaoqing Wang, Qianhong Liu, Peng Zhang

**Affiliations:** 1 School of Economics and Management, Weifang University of Science and Technology, Weifang, China; 2 Department of Economic Management, Shandong Foreign Trade Vocational College, Qingdao, China; Alexandru Ioan Cuza University: Universitatea Alexandru Ioan Cuza, ROMANIA

## Abstract

Entrepreneurial intention is widely recognized as a key precursor of entrepreneurial behaviour, yet existing research has more often examined personal entrepreneurial characteristics and perceived environmental support separately, with less attention to how they jointly shape intention and how entrepreneurial role models may condition these relationships. Guided primarily by social cognitive theory and complemented by the Theory of Planned Behavior, this study develops an integrated model to examine how entrepreneurial spirit, reflected in innovativeness and proactiveness, and entrepreneurial environment perception, reflected in perceived access to finance, perceived entrepreneurial education, and perceived policy support, are related to college students’ entrepreneurial intention, and whether these relationships are strengthened by entrepreneurial role models. Using survey data from 1,389 students in higher education institutions in Shandong Province, China, the hypotheses were tested through confirmatory factor analysis, structural equation modelling, and hierarchical regression. The results show that both entrepreneurial spirit and entrepreneurial environment perception are positively associated with entrepreneurial intention. In addition, entrepreneurial role models significantly strengthen the relationships between entrepreneurial spirit and entrepreneurial intention, and between entrepreneurial environment perception and entrepreneurial intention. These findings contribute to entrepreneurship intention research by showing that entrepreneurial intention is shaped not only by person-related dispositions and perceived support conditions, but also by role-model-based social learning that makes entrepreneurial pathways more credible and attainable for students.

## 1. Introduction

In the context of a rapidly changing global and Chinese economy, entrepreneurship has become a key engine for job creation, innovation, and sustainable development. Recent studies consistently show that students’ entrepreneurial intentions are closely linked to future start-up activities and can help mitigate youth unemployment and underemployment [1–3]. In China, the national strategy of “mass entrepreneurship and innovation” has further elevated entrepreneurship to a strategic priority for economic transformation and upgrading, encouraging universities to cultivate new generations of opportunity-oriented, innovation-driven graduates [4]. Against the background of intensified job market competition, China’s college graduates (the term “college students” is used here to refer to students in higher education, including both four-year undergraduate and three-year higher vocational programmes in China) are expected to reach 12.22 million in 2025, making it increasingly important to understand whether students view entrepreneurship as a realistic career option for easing employment pressure and leveraging new business opportunities in the digital and technological economy [5,6]. Yet, despite the expansion of entrepreneurship education, incubators, start-up parks and loan subsidy schemes, only a small proportion of college students actually attempt to start a business, and even fewer manage to sustain their ventures successfully. This paradox underscores the urgent need to better understand what drives or inhibits college students’ entrepreneurial intention in specific social and institutional contexts, so that existing policy and educational efforts can be translated into more effective and targeted interventions.

Entrepreneurial intention is a critical psychological precursor of entrepreneurial behaviour because it reflects an individual’s conscious willingness to start a business and is one of the best predictors of subsequent entrepreneurial action [1,7]. In recent years, a growing body of research has examined its antecedents, highlighting the roles of individual dispositions, perceptions of the environment, and cognitive evaluations [4,5]. At the individual level, traits and capacities such as entrepreneurial passion, risk-taking, innovativeness, proactiveness and achievement motivation have been shown to shape students’ attitudes towards entrepreneurship and their perceived behavioral control [8–10]. At the contextual level, entrepreneurship education, policy support, access to finance, and the broader cultural and institutional environment can either encourage or discourage students from considering entrepreneurship as a realistic career option [2,3,11]. In addition, recent empirical work on Chinese college students suggests that perceived entrepreneurial environment and university support mechanisms exert significant influence on entrepreneurial intention through psychological mechanisms such as self-efficacy, achievement motivation and professional identity [5,12]. In parallel, recent research on entrepreneurial universities has examined how institutional input, strategic planning, and staged support processes shape undergraduates’ entrepreneurial behaviour and career development, further underscoring the critical role of university-level support for student entrepreneurship [13]. However, although this literature is extensive, it remains fragmented in two important respects. First, prior studies have often examined internal entrepreneurial characteristics and perceived environmental support separately, rather than explaining how they jointly shape entrepreneurial intention within a single framework. Second, although entrepreneurial role models have been widely discussed in entrepreneurship research, they have been treated mainly as direct antecedents or background influences, while their potential role in conditioning how personal dispositions and environmental perceptions are translated into entrepreneurial intention remains insufficiently theorized and tested.

This study focuses on students enrolled in higher education institutions in Shandong Province, an economically developed coastal province in eastern China with a large population of university and higher vocational students and a strong manufacturing and service base. In recent years, Shandong has actively responded to the national strategy of “mass entrepreneurship and innovation” by expanding entrepreneurship education, incubators and financial support programmes for young people. At the same time, survey data and recent discussions suggest that many young people in Shandong tend to prioritize postgraduate entrance examinations and civil service or public institution recruitment over starting their own businesses, reflecting a strong preference for stable, secure careers and a relatively weak inclination towards entrepreneurship [3,14]. This context is theoretically informative rather than merely descriptive. In a setting where stable career pathways are often perceived as more attractive than entrepreneurial careers, the effects of entrepreneurial spirit and entrepreneurial environment perception on intention may be less automatic, and the motivational influence of entrepreneurial role models may become especially salient.

Although numerous studies have reported a positive relationship between entrepreneurial spirit and entrepreneurial intention, the evidence is not entirely consistent. Huang, An [15], for example, found that entrepreneurial awareness, opportunity perception, and entrepreneurial self-efficacy did not have significant direct effects on college students’ entrepreneurial intention. Dinis, do Paco [16] likewise reported that personal characteristics such as tolerance, ambiguity, locus of control, and innovativeness were not statistically significant predictors. In addition, Aydin, Knezović [17] found a negative association between proactiveness and entrepreneurial intention in a specific context. These findings suggest that the effect of entrepreneurial spirit may depend on contextual or methodological conditions rather than operating uniformly across settings. A similar pattern appears in the literature on the entrepreneurial environment. Although supportive conditions are generally associated with stronger entrepreneurial intention, some studies suggest that environmental perceptions may influence intention indirectly through mechanisms such as self-efficacy or achievement motivation [18], and individuals may still refrain from entrepreneurship because of uncertainty, risk, or competing career options [19–21]. Taken together, these mixed findings suggest that entrepreneurial intention cannot be fully explained by either internal dispositions or environmental perceptions alone.

Building on existing research, some scholars have begun to focus on the impact of entrepreneurial role models on entrepreneurial intention (e.g., [22–24]). Studies have found that entrepreneurial role models can be significant in stimulating entrepreneurial intention [25]. According to social cognitive theory, individuals can acquire entrepreneurial knowledge not only through direct learning but also by observing the behaviors of entrepreneurial role models [26]. Through their experiences and stories, entrepreneurial role models provide behavioral examples and reveal the possibilities and challenges of the entrepreneurial environment [24]. Accordingly, role models may do more than directly stimulate entrepreneurial intention, they may also strengthen the translation of entrepreneurial spirit into intention by providing visible behavioral scripts and strengthen the effect of entrepreneurial environment perception by making supportive conditions appear more credible, attainable, and actionable. Conceptually, the present study is primarily grounded in social cognitive theory, and further informed by the Theory of Planned Behavior. In our model, entrepreneurial spirit is treated as a person-related disposition, entrepreneurial environment perception reflects students’ subjective evaluations of external support conditions such as policy, finance and education, and entrepreneurial role models provide vicarious learning experiences that shape efficacy beliefs and outcome expectations. The Theory of Planned Behavior is used in a complementary way to clarify how these person- and context-related antecedents map onto attitudes towards entrepreneurship, perceived behavioral control and perceived social norms. Thus, the study develops a social cognitive account of entrepreneurial intention in which role models moderate the effects of entrepreneurial spirit and entrepreneurial environment perception.

Against this background, this study examines how entrepreneurial spirit and entrepreneurial environment perception jointly influence entrepreneurial intention among higher education students in Shandong Province and whether entrepreneurial role models moderate these relationships. Specifically, we address the following research questions: [1] Do entrepreneurial role models moderate the relationship between entrepreneurial spirit and entrepreneurial intention? [2] Do entrepreneurial role models moderate the relationship between entrepreneurial environment perception and entrepreneurial intention? By answering these questions, the study contributes to the entrepreneurship literature in several interrelated ways. First, it integrates entrepreneurial spirit and entrepreneurial environment perception into a single framework of entrepreneurial intention formation, rather than examining them as isolated antecedents. Second, it conceptualizes entrepreneurial role models as moderators rather than merely direct predictors, thereby clarifying their role as a contextual condition under which internal dispositions and environmental perceptions translate into entrepreneurial intention. Third, by testing the model among higher education students in Shandong Province, the study provides evidence from a non-Western regional ecosystem in which entrepreneurship competes with culturally valued secure career options, while also offering implications for entrepreneurship education and policy in China.

## 2. Literature review and hypothesis

Based on social cognitive theory and the theory of planned behavior, this study develops a conceptual framework that links entrepreneurial spirit, entrepreneurial environment perception, entrepreneurial role models and entrepreneurial intention. The following subsections critically synthesize prior research on these constructs and clarify the theoretical relationships among them and develop the hypotheses.

### 2.1. Entrepreneurial intention

Entrepreneurial intention occupies a central position in entrepreneurship research because it is widely regarded as one of the most immediate and robust predictors of entrepreneurial behaviour. Bird [27] first introduced the concept of entrepreneurial intention, defining it as a psychological construct that guides entrepreneurs to direct cognitive resources, energy, and behavior toward specific entrepreneurial goals. Robinson, Stimpson [28] argued that entrepreneurial intention serves as an important bridge through which individual and social factors are translated into entrepreneurial behaviour. Krueger Jr, Reilly [29] further emphasized its role as the core indicator for predicting whether an individual will engage in entrepreneurial activities. Taken together, these studies suggest that entrepreneurial intention is not a simple preference for self-employment, but a cognitively formed commitment shaped by how individuals evaluate entrepreneurship as desirable and feasible.

Existing research has delved into the multifaceted factors influencing entrepreneurial intention from both individual and environmental perspectives [1,30,31]. At the individual level, studies have examined traits, motivations, and forms of personal capital that shape entrepreneurial judgement and opportunity evaluation. Regarding individual traits, characteristics like risk propensity, achievement motivation, proactiveness, and innovativeness have significant facilitating or inhibiting effects on entrepreneurs’ ability to identify and seize entrepreneurial opportunities [7,32,33]. On the motivation dimension, intrinsic motivations such as dissatisfaction with existing products or services, the passion for tackling challenges, and personal beliefs in success, as well as extrinsic motivations like social networks, expected economic benefits, and community recognition, all exert varying degrees of driving or moderating effects on entrepreneurs’ entrepreneurial intention [34,35]. The individual capital domain includes prior knowledge, education level, and networking abilities, which, by enhancing an entrepreneur’s innovative performance and the quality of entrepreneurial activities, have a positive shaping effect on their entrepreneurial intention [36–38]. On the environmental level, research focuses on cultural atmosphere, market environment, family environment, and community ecology [39–41]. These studies collectively indicate that entrepreneurial intention is shaped by both internal dispositions and external conditions. However, this literature also remains somewhat fragmented, because personal and environmental influences are often examined separately rather than as interrelated components of intention formation. Entrepreneurial decisions are rarely produced by traits or environments alone; rather, they emerge from how individuals interpret their own capabilities and the supportiveness of the contexts in which they are embedded.

### 2.2. Entrepreneurial spirit and entrepreneurial intention

Entrepreneurial spirit is a multidimensional concept that reflects an individual’s tendency to respond to opportunities, uncertainty, and change in entrepreneurial ways. Prior research conceptualizes it as involving a cluster of orientations and capabilities related to innovation, initiative, creativity, and forward-looking action [42–45]. Rather than treating entrepreneurial spirit as a fixed personality trait, recent studies increasingly view it as a developable set of attitudes and behavioral tendencies that can be cultivated through education, learning, and practice [46].

Based on previous research, this study identifies innovativeness and proactiveness as key elements of entrepreneurial spirit among college students. Innovativeness refers to the willingness to break away from established technologies or practices and engage in creative solutions, novelty, experimentation, and new ideas. Proactiveness refers to the readiness to act in advance of potential problems, customer needs, or changes in the business environment, and is widely regarded as an important factor for entrepreneurial success [47]. For college students, these two dimensions are especially relevant because they capture both the cognitive orientation to generate novel ideas and the behavioral tendency to act on perceived opportunities. Together, they represent complementary aspects of entrepreneurial spirit that are theoretically well suited to explaining entrepreneurial intention [48].

Prior research generally supports a positive relationship between entrepreneurial spirit and entrepreneurial intention. Students with stronger innovativeness are more likely to recognize opportunities and view entrepreneurship as feasible, while students with stronger proactiveness are more likely to act on perceived opportunities and create favourable conditions for entrepreneurial action [49–51]. At the same time, the literature is not entirely consistent. Some studies have reported non-significant or even negative associations between certain dimensions of entrepreneurial spirit and entrepreneurial intention in specific contexts [15–17], suggesting that its effect may depend on context and on how entrepreneurial action is cognitively evaluated. Even so, the dominant body of theory and evidence continues to support a positive relationship. From the perspective of social cognitive theory, students with stronger innovativeness and proactiveness are more likely to perceive themselves as capable of recognizing opportunities, coping with challenges, and generating desirable outcomes. From the perspective of the theory of planned behavior, these qualities are also likely to foster more favourable attitudes toward entrepreneurship and stronger perceived behavioral control. Accordingly, entrepreneurial spirit is expected to strengthen entrepreneurial intention among college students. Based on the above, the following hypothesis is proposed:

*H1: Entrepreneurial spirit positively influences college students’ entrepreneurial intention.*

### 2.3. Entrepreneurial environment perception and entrepreneurial intention

Entrepreneurial environment refers to a set of external factors that collectively influence entrepreneurial activities, either facilitating or hindering them, thus affecting the perceived cost/benefit ratio of new venture creation [52]. The entrepreneurial environment includes not only direct support resources such as funding schemes, technical consulting, and market opportunities [53], but also indirect influencing factors such as policies [54], culture [55], and social networks [8,56]. For the present study, however, the focus is not on the entrepreneurial environment as an objective macro-level system, but on students’ subjective perception of whether relevant entrepreneurial support is available, accessible, and useful to them. This distinction is important because individuals do not respond directly to environmental conditions as such; rather, they respond to how those conditions are interpreted through their own experience, knowledge, and expectations. Accordingly, entrepreneurial environment perception is defined here as students’ subjective evaluation of the extent to which their surrounding environment provides conditions that are conducive to entrepreneurship.

Drawing on previous studies, such as Nguyen [57], Luo, Guo [58] and Nowiński, Haddoud [59], this study categorizes entrepreneurial environment perception into three dimensions: perceived access to finance, perceived entrepreneurial education, and perceived policy support. Although the broader entrepreneurship literature also discusses dimensions such as infrastructure, cultural support, and market opportunities, the present study focuses on these three dimensions because they are the forms of support most directly perceived by higher education students in the early stage of intention formation. For student entrepreneurs who typically lack established business networks, market power, and prior entrepreneurial experience, perceptions of finance, education, and policy support are especially salient because they shape whether entrepreneurship appears feasible at all. Rusu, Roman [60] emphasize that funding is a critical resource for entrepreneurial success, and lack of financial support is one of the main obstacles in the early stages of entrepreneurship. Empirical evidence also shows that access to finance can enhance college students’ entrepreneurial intention and entrepreneurial capabilities [53,61]. Overall, these studies suggest that access to finance matters not only because it reduces resource constraints, but also because it increases the perceived feasibility of entrepreneurial action.

The entrepreneurial education support provided by universities enhances students’ entrepreneurial knowledge and skills and helps them understand the challenges they may encounter during the entrepreneurial process, thereby reducing uncertainty and strengthening entrepreneurial intention [62]. Moreover, the perceived effectiveness of entrepreneurial education has a substantial impact on students’ entrepreneurial spirit and intention [39]. Complementing this line of work, recent studies on entrepreneurial universities have shown that systematic university support structures can strengthen students’ entrepreneurial capabilities and increase the likelihood that they will engage in entrepreneurial activities [13]. For higher education students, entrepreneurial education is especially important because it translates an abstract career possibility into a more understandable and actionable pathway. When students perceive entrepreneurship education as relevant and practical, they are more likely to view entrepreneurship as something they can prepare for rather than merely admire from a distance.

College students, as one of the most promising groups of potential entrepreneurs, often abandon entrepreneurial ventures because of insufficient funding, limited financing channels, and weak policy support [15]. Entrepreneurial policies such as tax incentives, subsidies, and low-cost or interest-free loans can reduce entry barriers and enhance the attractiveness of entrepreneurship [63]. Empirical studies have also found positive associations between entrepreneurial policies and entrepreneurial intention among college students in China and Vietnam [15,64–66]. Perceived policy support therefore matters not only as a background institutional condition, but also as a signal that entrepreneurship is socially recognized, institutionally encouraged, and practically supported. For students with limited entrepreneurial experience, such signals can reduce uncertainty and increase confidence in the legitimacy and attainability of entrepreneurial action.

Even within the same entrepreneurial environment, individuals may form different perceptions because of differences in experience, values, knowledge, and sources of information. Prior research suggests that when individuals perceive the entrepreneurial environment as favorable, they are more likely to experience intrinsic motivation and stronger entrepreneurial intention [58]. Similarly, perceived support from parents, family, friends, and surrounding social networks has also been linked to stronger entrepreneurial intention [67]. Taken together, these studies suggest that perceived entrepreneurial environment influences entrepreneurial intention because it shapes how individuals evaluate the desirability and feasibility of entrepreneurship. From the perspective of social cognitive theory, supportive environmental perceptions strengthen positive outcome expectations and reduce perceived barriers to action. From the perspective of the theory of planned behavior, favourable perceptions of finance, education, and policy support are likely to enhance entrepreneurial attitudes, strengthen perceived behavioral control, and signal greater social legitimacy for entrepreneurship. Although some studies indicate that environmental support may also operate indirectly through mechanisms such as self-efficacy or intrinsic motivation, the dominant expectation remains that students who perceive a more supportive entrepreneurial environment will be more likely to form stronger entrepreneurial intention. Based on the above, the following hypothesis is proposed:

*H2: Entrepreneurial environment perception positively influences college students’ entrepreneurial intention.*

### 2.4 The moderating role of entrepreneurial role models

Role models are often seen as a way of motivating individuals to perform novel behaviors and inspire them to set ambitious goals. Within the social cognitive tradition, and particularly its social learning component, Gibson [68] stated that role models are cognitive objects representing social roles that possess certain attributes. Individuals perceive similarities between themselves and the role model in certain dimensions and aim to acquire the same attributes through learning and imitation. Thus, role models are common references who set examples for others and may inspire or motivate individuals to make specific decisions and achieve certain goals [69].

In entrepreneurship research, role models are especially important because they do not merely provide abstract encouragement; they embody concrete examples of entrepreneurial action, possibility, and achievement [70]. This study defines role models as individuals who influence the achievements, motivation, and goals of those pursuing a similar career path through their behaviors, accomplishments, or personal traits. They may be personally known individuals or more distant figures encountered through stories, media, or educational settings, but in either case they can shape how entrepreneurship is understood, evaluated, and imagined by potential entrepreneurs.

Numerous studies have shown that entrepreneurial role models influence entrepreneurial intentions and behaviors by shaping the attitudes and beliefs of potential entrepreneurs [71]. According to Bandura [26], individuals can learn not only through direct experience but also through observing others, making role models an important source of indirect learning. However, prior studies have focused mainly on whether entrepreneurial role models directly increase entrepreneurial intention, while paying less attention to whether they condition the strength of the relationships between other antecedents and intention. This distinction is important because role models may not function only as another independent predictor; they may also shape how strongly students convert their entrepreneurial dispositions and environmental perceptions into entrepreneurial intention.

When individuals observe and internalize the entrepreneurial spirit demonstrated by role models, they are more likely to view entrepreneurship as feasible and to emulate entrepreneurial behaviors. This suggests a moderating mechanism between entrepreneurial spirit and entrepreneurial intention. Students with strong entrepreneurial spirit may already possess innovativeness and proactiveness, but these dispositions do not always translate automatically into entrepreneurial intention. Exposure to entrepreneurial role models can provide behavioural scripts, social proof, and vicarious efficacy cues that make entrepreneurial action appear more concrete and attainable. In this way, role models help students see how innovative ideas and proactive tendencies can be enacted in real entrepreneurial contexts, thereby strengthening the extent to which entrepreneurial spirit is transformed into entrepreneurial intention. Laviolette, Radu Lefebvre [72] demonstrated that narrative entrepreneurial role models significantly enhance individuals’ self-efficacy and entrepreneurial intention. When individuals can identify with the entrepreneurial role model in the narrative and hold a positive attitude toward them, this identification and positive attitude enhance their self-efficacy, thereby stimulating their entrepreneurial intention. Fellnhofer [73] highlighted the importance of introducing role models into entrepreneurship education, emphasizing that role models effectively ignite individuals’ entrepreneurial passion, significantly positively affecting their entrepreneurial self-efficacy, which in turn boosts entrepreneurial intention. Moreover, Nowiński and Haddoud [22] employed fuzzy-set qualitative comparative analysis (fsQCA) and found that an inspirational role model, combined with entrepreneurial attitude and self-efficacy, significantly enhances an individual’s entrepreneurial intention. They pointed out that a positive entrepreneurial attitude and higher self-efficacy, under the influence of an inspirational role model, significantly increased Polish students’ entrepreneurial intention. Abbasianchavari and Moritz [74] further noted that the presence of entrepreneurial role models significantly influences individuals’ entrepreneurial intentions and behaviors, with this impact depending on the type of role model, the duration of exposure to the role model, and the surrounding environment.

Collectively, these studies indicate that role models can amplify entrepreneurial cognition by strengthening self-efficacy, inspiring identification, and increasing the perceived feasibility of entrepreneurial action. From the perspective of social cognitive theory, this occurs through vicarious learning and strengthened outcome expectations. From the perspective of the theory of planned behavior, role models may reinforce favourable entrepreneurial attitudes and perceived behavioral control. Accordingly, entrepreneurial role models are expected to strengthen the positive relationship between entrepreneurial spirit and entrepreneurial intention. Based on the above, the following hypothesis is proposed:

*H3: Entrepreneurial role models strengthen the positive relationship between entrepreneurial spirit and entrepreneurial intention among college students.*

Entrepreneurial role models, through their entrepreneurial experiences, provide individuals with key information about the entrepreneurial environment. This information helps individuals better understand and assess the environment they are in, thereby influencing their entrepreneurial intention. Drawing on a motivational perspective, Morgenroth, Ryan [70] argue that role models not only motivate individuals through behavioral demonstration but also foster trust and a positive evaluation of the entrepreneurial environment by representing “possibility” and “achievement”. Entrepreneurs also tend to identify more strongly with role models who share similar characteristics such as gender, nationality, and industry [75].

When individuals observe role models they identify with succeeding in the entrepreneurial environment, they are more likely to believe they too can succeed in a similar setting. This sense of trust prompts individuals to form a positive view of the entrepreneurial environment, thereby enhancing their entrepreneurial intention. Thus, entrepreneurial role models, through the transmission of positive entrepreneurial environment information, help individuals assess the environment optimistically. When individuals learn from role models’ entrepreneurial experiences, including how they overcame challenges and ultimately succeeded, they are more likely to believe that their own entrepreneurial environment is supportive and capable of providing necessary resources and opportunities. This optimistic view of the entrepreneurial environment increases individuals’ entrepreneurial intention. Importantly, this logic also implies a moderating effect.

Even when students perceive policy support, financing opportunities, and entrepreneurship education as favourable, these perceptions may remain abstract or weakly connected to action. Role models can make such environmental support more credible and actionable by showing how these resources are actually recognized, accessed, and used in practice. In this way, role models may help students translate a favourable view of the entrepreneurial environment into a stronger belief that entrepreneurship is realistically achievable for someone like themselves. This mechanism should be especially salient when students identify with the role model and interpret the role model’s experience as evidence that environmental support can be successfully mobilized. From a social cognitive perspective, this occurs because role models shape efficacy beliefs and outcome expectations. From a theory of planned behavior perspective, they may strengthen the effect of environmental perceptions on intention by reinforcing perceived feasibility and social legitimacy. Therefore, entrepreneurial role models are expected to strengthen the positive relationship between entrepreneurial environment perception and entrepreneurial intention. Based on the above, the following hypothesis is proposed:

*H4: Entrepreneurial role models strengthen the positive relationship between entrepreneurial environment perception and entrepreneurial intention among college students.*

## 3. Research Method

### 3.1. Research model

**Fig 1:**
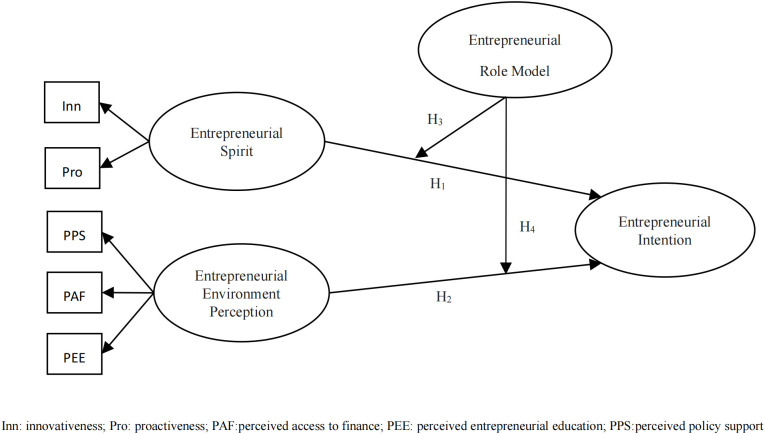
Research Model.

This study critically examined prior research and defined the core elements of entrepreneurial spirit among college students as innovativeness and proactiveness. It also subdivided the components of entrepreneurial environment perception into perceived access to finance, perceived entrepreneurial education, and perceived policy support. Aiming to verify how these aspects influence entrepreneurial intention and to explore the moderating role of entrepreneurial role models, we constructed a research model based on the interplay of these variables. The conceptual framework of the study is presented in Fig 1.

### 3.2. Variable Definition and Measurement

In order to ensure conceptual clarity and measurement rigour, all constructs in this study were measured using multi-item Likert-type scales that were adapted from established instruments and tailored to the context of Chinese higher education. Unless otherwise stated, all items were rated on a five-point scale ranging from 1 = “strongly disagree” to 5 = “strongly agree”. The full list of measurement items and their construct assignments is presented in S1 Table.

Entrepreneurial spirit was conceptualized as a second-order reflective construct with two first-order dimensions: innovativeness and proactiveness. The initial item pool was derived from Tu, Bhowmik [10], who developed a scale to capture college students’innovativeness and proactiveness in entrepreneurial activities. Based on their work and the specific context of Chinese innovation and entrepreneurship education, we retained and adapted five items for innovativeness and five items for proactiveness. The items for innovativeness tap students’ tendency to generate and prefer novel, unconventional entrepreneurial ideas and solutions, whereas the items for proactiveness capture the extent to which students take initiative and act ahead of others in identifying and responding to entrepreneurial opportunities and problems. In the measurement model, each first-order factor is indicated by its respective items, and the second-order ES factor is indicated by innovativeness and proactiveness.

Entrepreneurial environment perception was also modelled as a second-order reflective construct comprising three first-order dimensions, perceived policy support, perceived access to finance, and perceived entrepreneurial education. The measurement items for these dimensions were developed with reference to multiple studies in the entrepreneurship literature and were further refined to fit the Chinese policy environment, the university context, and the specific characteristics of college student entrepreneurship. More specifically, the items for perceived access to finance were developed by drawing on the relevant measures reported by Svotwa, Jaiyeoba [55] and Gianeta and Layman [76]; the items for perceived entrepreneurial education were developed with reference to Rahim and Mukhtar [75]; and the items for perceived policy support were developed by integrating insights from the institutional dimensions discussed by Busenitz, Gomez [77], as well as the policy-related measures reported by Meng, Shang [78]. In the context of higher education students, entrepreneurial environment perception is reflected primarily in how students evaluate the supportiveness of policy arrangements, the accessibility of financial resources, and the relevance of entrepreneurship education. These dimensions capture whether entrepreneurship is perceived as institutionally encouraged, financially feasible, and educationally supported at the intention-formation stage.

Perceived policy support reflects students’ perception of government policies and institutional arrangements favouring entrepreneurship, including the extent to which they believe entrepreneurship is encouraged and supported through relevant programmes, preferential measures, and institutional arrangements. This dimension is measured with seven items; perceived access to finance captures students’ perceived knowledge of, and access to, financial resources and channels suitable for student entrepreneurship, including business planning skills, familiarity with funding sources and perceived financial constraints. This dimension is measured with eight items; perceived entrepreneurial education assesses students’ evaluation of the quality and relevance of entrepreneurship education and related support offered by their institutions, including course content, teaching quality, practical activities and campus entrepreneurial climate. This dimension is measured with ten items. These three dimensions were retained because they jointly reflect whether students perceive entrepreneurship as institutionally supported, financially feasible, and educationally learnable. In other words, they capture key aspects of perceived support that are especially salient for students who are still in the pre-start-up stage.

Entrepreneurial intention was measured as a unidimensional reflective construct capturing students’ cognitive, attitudinal and behavioral willingness to start a business in the future. The four items were adapted from Wathanakom, Khlaisang [79], who developed and validated an entrepreneurial intention scale for university students. The items assess students’ intention, determination and current efforts to create their own business. Entrepreneurial role models were measured using four items adapted from Fellnhofer [73], who operationalised perceived role models in entrepreneurship education settings. The items capture students’ identification with, aspiration towards and emotional connection to a salient entrepreneurial role model.

All instruments were translated into Chinese and then back-translated into English by bilingual researchers to ensure semantic equivalence. Minor wording modifications were made after a pilot test with 80 students in Shandong Province to improve clarity and contextual relevance, while retaining the original meaning and dimensional structure of the scales.

### 3.3. Data collection procedure and sample characteristics

Data for this study were collected through an anonymous online questionnaire administered via Wenjuanxing, a widely used online survey platform in China. The initial version of the questionnaire was developed based on existing validated scales and the research context of this study. To assess the comprehensibility of the items and the reliability of the scales, a pilot test was conducted with 80 students from higher education institutions in Shandong Province. Based on the feedback obtained in the pilot, minor wording adjustments were made to several items to improve clarity and ensure that the questions were easy to understand. The final questionnaire began with an information page that explained the purpose of the study, stated that participation was voluntary, and assured respondents that their answers would be anonymous and used only for academic research. Only students who confirmed their willingness to participate could proceed to the main part of the survey.

During the formal survey, we adopted a cluster sampling approach, taking colleges or departments within higher education institutions as the sampling units. The sample was drawn from ten universities and higher vocational colleges located in three cities in Shandong Province, namely Qingdao, Yantai and Weifang. With the assistance of course instructors in the selected colleges or departments, the survey was administered in classrooms during short breaks between classes. The instructor briefly introduced the survey and invited students to participate. The Wenjuanxing quick response code and survey link were projected on the classroom screen, and students used their mobile phones to scan the code and fill in the questionnaire on site. Only full-time students who were currently enrolled and present in class at the time of the survey were invited to participate. Students could complete the questionnaire at their own pace within the break time, and they were informed that they could decline to participate or stop answering at any point without any negative consequences.

The online survey was carried out between 8 May 2025 and 7 June 2025. A total of 1,572 responses were collected. To ensure data quality, we removed questionnaires submitted from duplicate IP addresses and those in which all items for the main variables had the same response option. After this screening process, 1,389 valid questionnaires remained, resulting in an effective response rate of 88.36%. Subsequent analyses are based on this final sample.

In terms of the gender of the respondents, there were 628 males, accounting for 45.2%, and 761 females, accounting for 55.8%. Regarding the distribution of academic years, the third-year students were the most numerous, with 449 people, accounting for 32.3%, followed by fourth-year students, with 434 people, accounting for 31.2%. Due to the greater demands and concerns of third- and fourth-year students in terms of course schedules and internship opportunities, they participated in this survey more than first- and second-year students. In terms of educational level, this survey included 882 undergraduate students (63.5%) and 507 vocational students (36.5%). In terms of major distribution, the largest proportion was business and economics majors, with 576 people, as high as 41.5%, followed by science and engineering majors, with 332 people, accounting for 23.9%.

### 3.4. Ethical Statement

This study involved an anonymous questionnaire survey of adult participants. Because the study was non-interventional, minimal risk, and collected no identifiable information, it did not undergo formal ethics committee review. All participants were 18 years of age or older. Participation was voluntary, and informed consent was obtained electronically on the first page of the survey (participants clicked “agree/continue” before proceeding). Participants could stop at any time without penalty, and responses were analyzed only in aggregate.

## 4. Analysis Results

### 4.1. Common Method Variance

Given that the data collected in this study were obtained through a questionnaire survey completed independently by the respondents, there is a possibility that the respondents may subconsciously attempt to maintain consistency in their answers or provide ideal responses while answering the questionnaire questions. This could potentially lead to statistical measurement errors and result biases, or distort the true relationships between concepts, thereby causing common method bias, as discussed by Podsakoff, MacKenzie [80]. In view of this, we conducted a common method bias test before carrying out hypothesis testing. The Harman single-factor test results indicated that the contribution rate of the extracted maximum factor was 39.774%, which is below the threshold value of 50%. This suggests that there is no common method bias issue in this study [81].

### 4.2. Validity and Reliability analysis

A confirmatory factor analysis (CFA) was conducted in AMOS 26.0 to assess the measurement model. Entrepreneurial spirit was specified with two first-order dimensions (innovativeness and proactiveness), and entrepreneurial environment perception with three first-order dimensions (perceived policy support, perceived access to finance, and perceived entrepreneurial education), while entrepreneurial intention and role models were modelled as separate latent variables. As shown in Table 1, all standardized factor loadings on the intended constructs are significant and mostly above 0.60, and Cronbach’s alpha coefficients for the dimensions range from 0.71 to 0.96, indicating good internal consistency and convergent validity. The overall model fit is acceptable ($\chi^{\text{2}}$= 3085.210, df = 394, RMSEA = 0.070, IFI = 0.922, TLI = 0.914, CFI = 0.922), suggesting that the measurement model adequately represents the data.

**Table 1:** Results of confirmatory factor analysis for the measurement model.

Latent construct	Indicator	Standardized Loading	S.E.	C.R. (p-value)	Cronbach’s α
Entrepreneurial Spirit – Innovativeness (Inn)	Inn4	0.870	–	–	0.714
	Inn3	0.641	0.033	20.522***	
Entrepreneurial Spirit – Proactiveness(Pro)	Pro4	0.691	0.045	22.790***	0.768
	Pro3	0.755	0.044	24.511***	
	Pro1	0.728	–	–	
Entrepreneurial environment perception – Perceived policy support (PPS)	PPS7	0.797	–	–	0.961
	PPS6	0.926	0.028	42.508***	
	PPS5	0.932	0.028	42.925***	
	PPS4	0.923	0.028	42.265***	
	PPS3	0.905	0.029	41.034***	
	PPS2	0.856	0.031	37.776***	
	PPS1	0.835	0.030	36.437***	
Entrepreneurial environment perception – Perceived access to finance (PAF)	PAF8	0.793	–	–	0.923
	PAF7	0.806	0.032	33.461***	
	PAF6	0.814	0.028	33.918***	
	PAF5	0.872	0.031	37.205***	
	PAF4	0.846	0.030	35.676***	
	PAF3	0.769	0.029	31.475***	
Entrepreneurial environment perception – Perceived entrepreneurial education (PEE)	PEE9	0.690	0.037	26.543***	0.923
	PEE8	0.913	0.030	36.793***	
	PEE7	0.916	0.030	36.939***	
	PEE6	0.834	0.029	33.030***	
	PEE5	0.824	0.032	32.545***	
	PEE2	0.755	–	–	
Entrepreneurial Intention (EI)	EI1	0.849	–	–	0.887
	EI2	0.817	0.025	35.611***	
	EI3	0.813	0.029	35.415***	
	EI4	0.789	0.027	33.944***	
Role Models (RM)	RM1	0.773			0.817
	RM2	0.897	0.053	23.364***	

$\chi^{\text{2}}$ = 3085.210, df = 394, IFI = 0.922, TLI = 0.914, CFI = 0.922, RMSEA = 0.070

Based on the first-order results, a second-order CFA was conducted to test whether entrepreneurial spirit (ES) and entrepreneurial environment perception (EEP) can be treated as higher-order constructs. As shown in Table 2, innovativeness and proactiveness both load strongly and significantly on ES (0.842 and 0.949, p < 0.001), indicating that they reflect a common latent dimension of entrepreneurial spirit. Likewise, perceived policy support, perceived access to finance and perceived entrepreneurial education load substantially on EEP (0.794, 0.830 and 0.804, all p < 0.001), suggesting that they jointly capture students’ overall perception of the entrepreneurial environment.

**Table 2:** Second-order CFA: standardized loadings of first-order dimensions on higher-order constructs.

Higher-order construct	First-order dimension	Standardized loading	S.E.	C.R (p-value)	AVE	CR
Entrepreneurial Spirit (ES)	Inn	0.842	–	–	0.805	0.891
	Pro	0.949	0.051	19.164***		
Entrepreneurial environment perception (EEP)	PPS	0.794	–	–	0.655	0.851
	PAF	0.830	0.054	22.309***		
	PEE	0.804	0.057	21.397***		
Entrepreneurial Intention (EI)	EI1	0.849	–	–	0.668	0.889
	EI2	0.817	0.025	35.611***		
	EI3	0.813	0.029	35.415***		
	EI4	0.789	0.027	33.944***		
Role Models (RM)	RM1	0.773	–	–	0.701	0.823
	RM2	0.897	0.053	23.364***		

*Note:* [1] *All standardized loadings are significant at p < 0.001;* [2] *ES:Entrepreneurial Spirit, EEP:Entrepreneurial environment perception, EI:Entrepreneurial Intention, RM:Role Models*

The AVE values for ES and EEP are 0.805 and 0.655, and the composite reliability values are 0.891 and 0.851, all exceeding the usual benchmarks of 0.50 for AVE and 0.70 for composite reliability [82]. For entrepreneurial intention (EI) and role models (RM), AVE (0.668 and 0.701) and composite reliability (0.889 and 0.823) are also satisfactory. These results support the convergent validity and internal consistency of all latent constructs and provide empirical justification for modelling ES and EEP as higher-order factors in the structural analysis.

To further assess discriminant validity, we computed the heterotrait–monotrait (HTMT) ratios between the latent constructs. As reported in Table 3, the HTMT values range from 0.333 to 0.623. All ratios are well below the commonly recommended thresholds of 0.85 or 0.90 for establishing discriminant validity [84]. This indicates that entrepreneurial spirit (ES), entrepreneurial environment perception (EEP), entrepreneurial intention (EI) and role models (RM) are empirically distinguishable and that the measurement model demonstrates satisfactory discriminant validity.

**Table 3:** Discriminant validity using HTMT.

	ES	EEP	EI	RM
**ES**				
**EEP**	0.494			
**EI**	0.623	0.525		
**RM**	0.398	0.610	0.333	

*Note: ES:Entrepreneurial Spirit, EEP:Entrepreneurial environment perception, EI:Entrepreneurial Intention, RM:Role Models*

### 4.3. Hypothesis testing results

Based on the second-order measurement structure, we estimated a structural model in which entrepreneurial spirit (ES) and entrepreneurial environment perception (EEP) jointly predict entrepreneurial intention (EI). The overall model fit is acceptable, with $\chi^{\text{2}}$= 2806.978, df = 342, IFI = 0.925, TLI = 0.917, CFI = 0.925 and RMSEA = 0.072, which are all within the recommended ranges for a reasonably well-fitting structural model (Hair et al., 2017).

As shown in Table 4, entrepreneurial spirit has a significant positive effect on entrepreneurial intention (estimate = 0.727, S.E. = 0.055, C.R. = 13.274, p < 0.001), supporting H1. This result indicates that students who display stronger innovativeness and proactiveness tend to report higher levels of entrepreneurial intention. In other words, person-related dispositions that reflect a willingness to generate novel ideas and to act proactively are strongly associated with a greater willingness to start a business in the future.

**Table 4:** Hypothesis testing results.

Hypothesis	Path	Estimate	S.E.	C.R.	P	Decision
H1	EI ← ES	0.727	0.055	13.274	***	Supported
H2	EI ← EEP	0.493	0.057	8.613	***	Supported
$\chi^{\text{2}}$ *=2806.978, df = 342, IFI = 0.925, TLI = 0.917, CFI = 0.925, RMSEA = 0.072*						

Entrepreneurial environment perception also exerts a significant positive effect on entrepreneurial intention (estimate = 0.493, S.E. = 0.057, C.R. = 8.613, p < 0.001), supporting H2. This suggests that students who perceive higher levels of policy support, access to finance and entrepreneurial education are more likely to consider entrepreneurship as a viable career option. Comparing the two paths, the effect of entrepreneurial spirit on entrepreneurial intention is stronger than that of entrepreneurial environment perception, which implies that internal dispositions and external perceptions both matter, but that person-related factors play a more pronounced role in shaping entrepreneurial intention within this sample.

**Fig 2:**
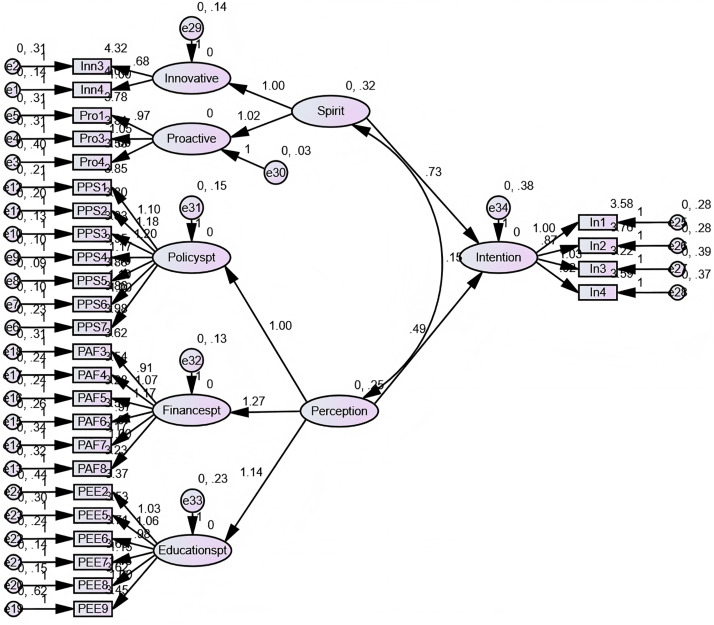
Structural model testing result.

To provide a more intuitive overview of these relationships, Fig 2 presents the structural path model used to test H1 and H2, including the path coefficients from ES and EEP to EI.

### 4.4. Moderating effect test results

To test H3, we used PROCESS (Model 1) with entrepreneurial intention (EI) as the dependent variable, entrepreneurial spirit (ES) as the predictor, role models (RM) as the moderator, and gender, year of study, education level and major as control variables. As shown in Table 5, both ES (B = 0.661, SE = 0.034, t = 19.670, p < 0.001, 95% CI [0.595, 0.727]) and RM (B = 0.143, SE = 0.032, t = 4.503, p < 0.001, 95% CI [0.081, 0.206]) have significant positive main effects on EI. More importantly, the interaction term ES × RM is positive and significant (B = 0.223, SE = 0.047, t = 4.798, p < 0.001, 95% CI [0.132, 0.315]), and the change in explained variance is significant (ΔR² = 0.011, p < 0.001). This result indicates that higher exposure to entrepreneurial role models strengthens the positive relationship between entrepreneurial spirit and entrepreneurial intention, which supports H3. Among the control variables, gender and year of study show significant effects on EI, whereas education level and major do not.

**Table 5:** Moderating effect of role models on the relationship between ES and EI.

Variable	Unstandardized coefficient		t	p	LLCI	ULCI	R^2^ change
	B	SE					
Constant	3.907	0.102	38.316	0.000	3.707	4.108	0.011***
ES	0.661	0.034	19.670	0.000	0.595	0.727	
RM	0.143	0.032	4.503	0.000	0.081	0.206	
ES × RM	0.223	0.047	4.798	0.000	0.132	0.315	
Gender	−0.175	0.040	−4.408	0.000	−0.253	−0.097	
Academic Year	−0.078	0.019	−4.010	0.000	−0.116	−0.040	
Education level	0.052	0.040	1.286	0.199	−0.027	0.131	
Major	0.009	0.012	0.753	0.450	−0.015	0.034	

Note: ***p < .001, **p < .01, *p < .05; ES: entrepreneurial spirit, RM: role model, EI: entrepreneurial intention

**Fig 3:**
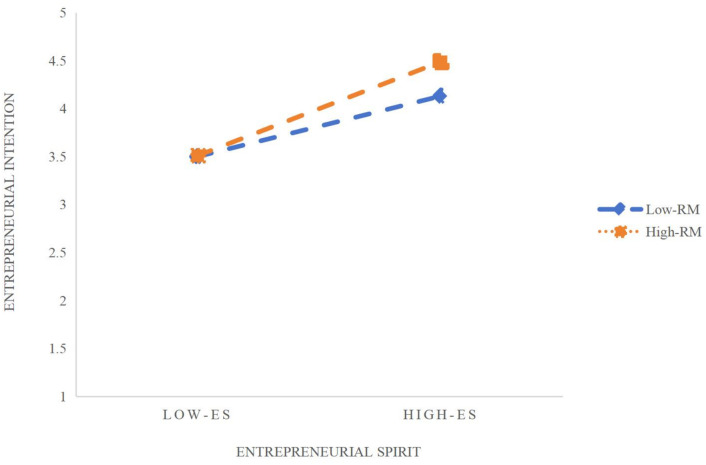
Moderating effect of role models on the relationship between ES and EI.

To more vividly illustrate the moderating effect of role models, this study presents a graphical representation. As shown in Fig 3, compared to the low role model condition (slope = 0.518, p < 0.001), in the high role model condition (slope = 0.805, p < 0.001), the positive effect of entrepreneurial spirit on entrepreneurial intention is stronger. Therefore, H3 is further validated.

For H4, as shown in Table 6, the moderation analysis with entrepreneurial environment perception (EEP) as the predictor and entrepreneurial intention as the outcome shows that EEP remains a significant positive predictor (B = 0.554, SE = 0.036, t = 15.560, p < 0.001). The main effect of role models (RM) is not significant, but the interaction term EEP × RM is positive and statistically significant (B = 0.173, SE = 0.043, t = 4.048, p < 0.001, 95% CI [0.089, 0.257]), and the associated change in explained variance is small but significant (ΔR² = 0.008, p < 0.001). This indicates that the positive relationship between EEP and EI is stronger for students who report higher exposure to entrepreneurial role models, which supports H4.

**Table 6:** Moderating effect of role models on the relationship between EEP and EI.

Variable	Unstandardized coefficient		t	p	LLCI	ULCI	R^2^ change
	B	SE					
Constant	3.968	0.1025	37.751	0.000	3.762	4.175	0.008***
EEP	0.554	0.036	15.560	0.000	0.484	0.624	
RM	0.049	0.037	1.317	0.188	−0.024	0.121	
EEP × RM	0.173	0.043	4.048	0.000	0.089	0.257	
Gender	−0.290	0.040	−7.199	0.000	−0.369	−0.211	
Academic Year	−0.052	0.020	−2.590	0.010	−0.091	−0.013	
Education level	0.051	0.041	1.242	0.241	−0.030	0.132	
Major	0.023	0.013	1.778	0.076	−0.002	0.048	

Note: ***p < .001, **p < .01, *p < .05; EEP: entrepreneurial environment perception, RM: role models, EI: entrepreneurial intention

**Fig 4:**
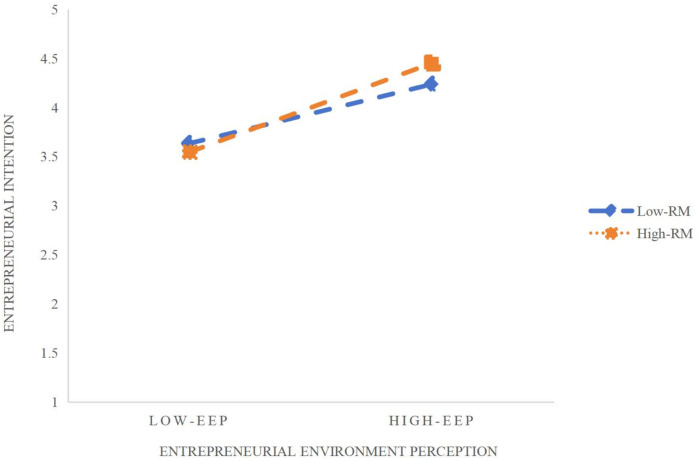
Moderating effect of role models on the relationship between EEP and EI.

As shown in Fig 4, compared to the low role model condition (slope = 0.443, p < 0.001), in the high role model condition (slope = 0.665, p < 0.001), the positive effect of entrepreneurial environment perception on entrepreneurial intention is stronger. Therefore, H4 is further validated.

## 5. Discussion and conclusions

Drawing on social cognitive theory and the theory of planned behavior, this study examined how entrepreneurial spirit and entrepreneurial environment perception are related to entrepreneurial intention among higher education students in Shandong Province, and how entrepreneurial role models shape these relationships. The findings support the view that entrepreneurial intention is not determined by a single factor, but emerges from the interplay between person related dispositions, subjective evaluations of the entrepreneurial context and socially transmitted experiences such as exposure to role models. The findings further suggest that entrepreneurial intention formation is a context-sensitive cognitive process in which internal dispositions and environmental perceptions are shaped by socially meaningful cues such as entrepreneurial role models. This interpretation extends beyond a simple trait-based account of entrepreneurship and highlights the importance of relational and contextual influences in higher education settings.

The results indicate that entrepreneurial spirit positively influences college students’ entrepreneurial intention. This finding is consistent with the views of Bazan, Gaultois [83] and Rumangkit and Hadi [46], who emphasized the importance of entrepreneurial spirit in stimulating entrepreneurial intention. In particular, the positive effects of innovativeness and proactiveness align with the empirical results show that college students’ innovativeness and proactiveness each positively influence their entrepreneurial intention. This aligns with the findings of Al-Mamary and Alshallaqi [33], who found a significant positive correlation between autonomy, innovativeness, risk-taking, proactiveness, and the entrepreneurial intention of Saudi Arabian university students. At the same time, our results should be interpreted against the backdrop of prior studies reporting non-significant or even negative effects of certain entrepreneurial traits in specific contexts. Rather than contradicting those studies, the present findings suggest that the relationship between entrepreneurial spirit and entrepreneurial intention may depend on whether students can interpret their innovativeness and proactiveness as relevant and actionable within their own career environment. In the present sample, entrepreneurial spirit appears to function as a meaningful personal resource rather than as a latent disposition disconnected from behavioural intention. From the perspective of social cognitive theory, innovativeness and proactiveness function as person related dispositions that shape how students interpret entrepreneurial opportunities, evaluate risks and form expectations about their ability to act successfully. In terms of the theory of planned behavior, innovativeness and proactiveness can be understood as antecedents that contribute to more favourable attitudes towards entrepreneurship and stronger perceived behavioral control, both of which are central determinants of entrepreneurial intention. This finding also suggests that entrepreneurial intention models should pay greater attention to how proactive and innovative tendencies are activated in specific educational contexts, rather than assuming that such traits operate uniformly across settings.

This study also reveals that perceptions of policy support, access to finance, and entrepreneurial education support all positively influence college students’ entrepreneurial intention. This result is consistent with prior empirical research. For example, Bazan, Gaultois [83] emphasized that government entrepreneurial policies can enhance entrepreneurs’ confidence and increase the likelihood of their participation in entrepreneurial activities. Shahriar, Hassan [61], by extending the Theory of Planned Behavior, showed that access to financing has a significant positive impact on college students’ entrepreneurial intention and plays an important mediating role between attitude, subjective norms, perceived behavioral control, and entrepreneurial intention. Rusu, Roman [60] also found that access to finance is a crucial factor affecting youth entrepreneurial intention, with financial support from family and friends having a significant positive impact, and Svotwa, Jaiyeoba [53] empirically verified that perceived access to finance strengthens entrepreneurial capabilities, which in turn affect entrepreneurial intention. Su, Zhu [49] identified perceived entrepreneurial education support as an important determinant of Chinese college students’ entrepreneurial attitudes and perceived behavioral control, thereby influencing their entrepreneurial intention. However, the present findings also add nuance to the literature by showing that it is not merely the objective presence of support that matters, but students’ subjective interpretation of whether such support is accessible and useful to them. Our results indicate that when students perceive policy, finance, and education support as meaningful and actionable, these environmental factors are more likely to function as intention-enhancing conditions. Social cognitive theory highlights that individuals do not respond to the objective environment as such, but to their cognitive appraisal of environmental opportunities and constraints. When students perceive that policies are supportive, that financial resources are accessible and that their university offers relevant entrepreneurial education, they are more likely to judge entrepreneurship as feasible and worthwhile. In terms of the theory of planned behavior, these perceptions contribute to more positive attitudes towards starting a business, stronger perceived behavioral control and more favourable subjective norms, since supportive policies, financing channels and educational programmes signal that entrepreneurship is socially valued and institutionally enabled. The significance of all three dimensions therefore supports a more integrated view of entrepreneurial intention formation in which policy, finance, and education jointly shape how students evaluate entrepreneurship as a realistic career path.

The results also reveal that entrepreneurial role models play a moderating role in the relationships between entrepreneurial spirit and entrepreneurial intention, as well as between entrepreneurial environment perception and entrepreneurial intention. This finding is consistent with previous empirical studies but also deepens the theoretical understanding of how role models operate in the entrepreneurial process. For example, Abbasianchavari and Moritz [23] pointed out that entrepreneurial role models promote entrepreneurial behavior mainly by enhancing individuals’ self-efficacy, while our study shows that when students have higher exposure to such role models, the positive effects of innovativeness and proactiveness on entrepreneurial intention become stronger. Similarly, Otache, Edopkolor [84] based on the Theory of Planned Behavior, indicated that entrepreneurial role models indirectly influence entrepreneurial intention by enhancing perceived behavioral control. Our results extend this line of work in two ways. First, they suggest that role models should not be understood only as direct antecedents or indirect psychological triggers, but also as boundary conditions that shape how strongly other antecedents are converted into entrepreneurial intention. Second, they show that the motivational influence of role models is relevant not only to person-related factors such as innovativeness and proactiveness, but also to contextual perceptions such as policy, finance, and educational support. In other words, role models help connect both person-related dispositions and contextual perceptions to entrepreneurial intention.

From the perspective of social cognitive theory, these moderating effects can be understood as the outcome of vicarious learning through the observation of others’ behaviour and outcomes. Exposure to entrepreneurial role models provides concrete examples of how innovative and proactive tendencies can be translated into entrepreneurial action and how supportive elements of the entrepreneurial environment can be mobilized in practice. When students identify with such role models, their efficacy beliefs and outcome expectations are more likely to be strengthened, which amplifies the extent to which their own entrepreneurial spirit and environmental perceptions are transformed into intention. In terms of the theory of planned behavior, role models can also reinforce positive attitudes towards entrepreneurship and perceived social approval by embodying successful or socially valued entrepreneurial careers. The moderating effects found in this study therefore suggest that entrepreneurial role models occupy a more central theoretical position than is often assumed in entrepreneurship intention research. Rather than simply adding another predictor to the model, they help explain why similar levels of entrepreneurial spirit or environmental support do not always produce the same level of entrepreneurial intention across students. This insight is especially relevant in contexts such as Shandong, where entrepreneurship coexists with strong preferences for secure and institutionally valued career paths. In such settings, role models may function as legitimizing and activating cues that make entrepreneurship appear not only possible, but personally attainable. More broadly, the findings suggest that entrepreneurial intention in higher education depends not only on individual capability and environmental support, but also on whether students can place those factors within a socially believable pathway to action.

Overall, this study contributes to the literature by showing that entrepreneurial intention among higher education students is shaped by the interaction of personal dispositions, perceived support conditions, and role-model-based social learning. Although the empirical setting is Shandong Province, the theoretical implication is broader. In higher education contexts where students face competing career scripts and uncertainty about entrepreneurial feasibility, entrepreneurial role models may be especially important in bridging the gap between favourable dispositions or perceptions and concrete entrepreneurial intention.

## 6. Theoretical and practical implications

The findings of this study provide several theoretical implications for entrepreneurship research. First, by jointly examining entrepreneurial spirit, entrepreneurial environment perception, and entrepreneurial role models, the study advances a more integrated explanation of entrepreneurial intention formation among higher education students. The positive associations between innovativeness, proactiveness and entrepreneurial intention, as well as between perceived policy support, perceived access to finance, perceived entrepreneurial education and entrepreneurial intention, are consistent with the idea that intention is shaped by individuals’ cognitive evaluations of opportunities, risks and their own capabilities rather than by stable personality traits alone. This person–environment pattern also resonates with the Theory of Planned Behavior, in which attitudes towards entrepreneurship, perceived behavioral control and subjective norms are key determinants of entrepreneurial intention. Innovativeness and proactiveness, together with favourable perceptions of policy, finance and education, can be understood as antecedents that foster more positive attitudes, stronger perceived behavioral control and more supportive subjective norms, thereby reinforcing students’ willingness to engage in entrepreneurial action. Rather than treating entrepreneurial intention as the outcome of either internal traits or external support alone, the study shows that it emerges from the interaction between person-related dispositions and context-related perceptions in higher education settings.

The study also refines the theoretical understanding of entrepreneurial role models in the entrepreneurship literature. Prior research has predominantly treated role models as independent predictors of entrepreneurial intention or as sources of indirect influence through mediating mechanisms such as self efficacy and entrepreneurial capabilities. In contrast, the present study conceptualizes and empirically validates entrepreneurial role models as a moderating factor that shapes how entrepreneurial spirit and entrepreneurial environment perception are translated into intention. This suggests that role models do not merely add another direct pathway to intention, but condition the strength of the links between person related dispositions, contextual perceptions and entrepreneurial intention. From a social cognitive perspective, this highlights the importance of vicarious learning and identification with role models in amplifying or attenuating the impact of individual and environmental factors. From a planned behaviour perspective, it implies that role models may reinforce perceived social approval for entrepreneurship and strengthen the process through which attitudes and perceived control are transformed into intention. In other words, role models help explain why students with similar levels of entrepreneurial spirit or similarly favourable environmental perceptions may nevertheless differ in their entrepreneurial intention. By identifying entrepreneurial role models as contextual moderators, the study moves the literature beyond direct-effect and mediation-based explanations and offers a more differentiated understanding of entrepreneurial intention formation.

At the same time, our use of the Theory of Planned Behavior is primarily complementary. By illustrating how innovativeness, proactiveness and perceived policy, finance and education support can be mapped onto attitudes, perceived behavioral control and subjective norms, the study refines the TPB account of entrepreneurial intention while leaving its core assumptions intact. Accordingly, the study does not seek to replace TPB, but to specify more clearly which personal and contextual factors may feed into its core components in the context of student entrepreneurship. This also helps connect entrepreneurship intention research to broader intention-based theorizing.

In terms of practical implications, the findings suggest that efforts to strengthen college students’ entrepreneurial intention should address personal dispositions, perceived support conditions, and role-model-based social learning at the same time. Universities should prioritize fostering innovative thinking by enhancing students’ innovation capabilities through curriculum design and practical activities. Collaboration with businesses to establish innovation labs can also provide students with opportunities to engage with and learn cutting-edge technologies and ideas. Furthermore, universities should work to create a campus culture that encourages innovation, such as regularly organizing innovation result exhibitions and lectures, and establishing innovation scholarships to motivate students to actively participate in innovation practices. To enhance students’ entrepreneurial proactiveness, universities can offer career planning courses and targeted guidance to help students clarify their entrepreneurial direction, strengths, and opportunities. A variety of entrepreneurial practice activities, such as entrepreneurship simulation competitions and project incubation, should be organized, and entrepreneurship practice bases should be established to provide students with venues and resources for simulating entrepreneurship. These initiatives are important because the results indicate that entrepreneurial intention is strengthened not only by creative thinking but also by action-oriented tendencies such as proactiveness.

Universities can also help students better understand current entrepreneurial policies by organizing lectures and information exchange sessions while offering training in business-plan writing and financing techniques, and collaborating with enterprises to provide more accessible financing channels. Entrepreneurial education support can be achieved by developing a comprehensive curriculum that includes entrepreneurial knowledge, practical skills, and psychological resilience, while also strengthening faculty development and providing more practical platforms, such as entrepreneurship incubators and co-working spaces. At the policy and institutional level, support measures should be designed not only to exist objectively, but also to be visible, understandable, and usable from the student perspective. In practical terms, this means improving the communication of entrepreneurship policies, creating clearer entry points to financing channels, and offering entrepreneurship education that students perceive as relevant to real venture creation rather than as symbolic or purely theoretical training.

Given the motivational role of entrepreneurial role models, diverse role models should be introduced in entrepreneurship education, encompassing entrepreneurs from different genders, industry backgrounds, and experiences of both success and failure. Opportunities for sustained interaction with such role models through mentorship programmes, entrepreneur talks, and alumni networks can help students build stronger entrepreneurial identity and more positive entrepreneurial cognition. Because role models were found to strengthen the effects of both entrepreneurial spirit and entrepreneurial environment perception, role model exposure should be integrated into entrepreneurship support systems rather than treated as a supplementary activity. The practical value of such initiatives lies in making entrepreneurship appear not only desirable, but also socially credible and personally attainable.

## 7. Limitations and suggestions for future studies

This study empirically validates the relationship between entrepreneurial spirit, perceptions of the entrepreneurial environment, and entrepreneurial intention, while also exploring the moderating role of entrepreneurial role models. The findings provide new perspectives and theoretical support for research in the field of entrepreneurship. However, there are several limitations in the study, which offer directions for further improvement and development in future research.

This study’s sample is limited to college students from several universities and higher vocational colleges in Shandong Province. While this sampling frame facilitates data collection and allows for a relatively large sample size, it inevitably limits the external validity and generalisability of the results. The participating institutions are all located in one province and, although they include both universities and higher vocational colleges as well as business-related and science and engineering disciplines, they do not cover the full diversity of provincial contexts, disciplinary profiles or institutional types that exist within China or across countries. The college student group also has unique characteristics in terms of access to entrepreneurial resources, entrepreneurial education backgrounds and personal development needs, and the mechanisms behind their entrepreneurial intention formation may differ significantly from other groups. Future research could expand to a broader population, including individuals from different provinces, age groups, professional backgrounds, institutional types and cultural contexts. This would allow for a more comprehensive verification of the universality of the moderating role of entrepreneurial role models, reveal the heterogeneity of the relationship between entrepreneurial spirit, perceptions of the entrepreneurial environment and entrepreneurial intention in different groups, and provide more targeted recommendations for entrepreneurship education and policy-making.

This study uses cross-sectional data, which only reflects the relationships between variables at a specific point in time and cannot capture the dynamic changes between entrepreneurial spirit, perceptions of the entrepreneurial environment, entrepreneurial role models, and entrepreneurial intention. Entrepreneurial intention is a dynamic psychological process that may be continuously influenced by various factors, including individual growth, environmental changes, and social interactions. Therefore, future research could adopt a longitudinal study design to track changes in individuals at different stages after interacting with entrepreneurial role models, observing the dynamic evolution of entrepreneurial spirit, entrepreneurial environment perception, and entrepreneurial intention. Such designs would make it possible to examine more clearly how entrepreneurial intention develops over time and whether the moderating role of entrepreneurial role models changes across different stages of entrepreneurial cognition and career development.

Another limitation concerns the scope of construct operationalisation. In this study, entrepreneurial environment perception was captured through perceived policy support, perceived access to finance, and perceived entrepreneurial education, which are especially salient to higher education students at the entrepreneurial intention stage. Although this operationalisation is appropriate for the student context, it does not encompass all possible dimensions of the broader entrepreneurial environment. Future research could extend this framework by incorporating additional dimensions such as perceived market opportunities, cultural support for entrepreneurship, infrastructure and business services, or regional entrepreneurial climate. Doing so would help provide a more comprehensive understanding of how different layers of the entrepreneurial environment shape intention formation.

In addition, although this study explores the moderating role of entrepreneurial role models, the analysis of their underlying mechanisms remains relatively limited. The role of entrepreneurial role models in different contexts may vary significantly due to factors such as industry, cultural background, and social networks. Future research could further investigate the diverse mechanisms of entrepreneurial role models in various contexts. For example, future studies could examine whether role models operate mainly by enhancing self-efficacy, strengthening identification, providing industry-specific information, increasing the perceived credibility of environmental support, or reducing the psychological uncertainty associated with entrepreneurship. Their role may also differ across industries, institutional settings, and cultural contexts. In addition, future research could further explore how entrepreneurial role models interact with other contextual factors, such as policy support, social networks, and local entrepreneurial culture, in shaping entrepreneurial intention. Such work would deepen understanding of the conditions under which role models are most influential and offer more fine-grained implications for entrepreneurship education and policy design.

In addition, all variables in this study were measured through self-reported perceptions in a single questionnaire at one point in time. This raises the possibility of common method bias and potential endogeneity, for example due to unobserved personality traits or prior entrepreneurial experiences that may simultaneously influence entrepreneurial spirit, entrepreneurial environment perception and entrepreneurial intention. Although we conducted statistical tests for common method bias and implemented procedural remedies, such as assuring anonymity and varying the order of items, these steps cannot completely rule out method-related or endogeneity problems. Future research could address these limitations by combining self-reports with behavioral or administrative data, introducing temporal separation between the measurement of predictors and outcomes, or employing longitudinal, experimental or quasi-experimental designs to better identify causal mechanisms.

## Supporting information

S1 Table Measurement items and construct assignments. Full list of measurement items used in this study and their construct assignments.
(DOCX)

S2 File De-identified minimal dataset. Respondent-level raw data and variable codebook used in this study.
(XLSX)
