# Supplementary material for: Linking entrepreneurial spirit and entrepreneurial environment perception to entrepreneurial intention: Moderation by entrepreneurial role models
Source: PLoS One. 2026 Jul 6;21(7):e0352807. doi: 10.1371/journal.pone.0352807 (PMC13336219; doi:10.1371/journal.pone.0352807)
Supplement: S1 Table — Full list of measurement items used in this study and their construct assignments. (DOCX) [file pone.0352807.s001.docx]

S1 Table. Measurement items and construct assignments

| **Construct Items** |
| --- |
| **Entrepreneurial Spirit** |
| ***- Proactiveness*** |
| Pro1. I am willing to be at the forefront in exploring entrepreneurial opportunities. |
| Pro2. I am able to identify potential entrepreneurial opportunities or problems ahead of others. |
| Pro3. I tend to take action earlier than others to address issues arising during entrepreneurship. |
| Pro4. I like to act quickly when facing entrepreneurial challenges or changes. |
| Pro5. Problems in the entrepreneurial process actively drive me to take necessary actions. |
| ***- Innovativeness*** |
| Inn1. I often like to try new, creative, or unconventional entrepreneurial ideas or activities. |
| Inn2. In entrepreneurial projects, I generally prefer a strong emphasis on uniqueness and novelty. |
| Inn3. When learning entrepreneurial knowledge or skills, I prefer to try my own unique way rather than following the crowd. |
| Inn4. I like to apply innovative and experimental approaches to solve entrepreneurial problems. |
| Inn5. I believe there are always new and better ways to ding things. |
| **Entrepreneurial Environment Perception** |
| ***- Perceived policy support*** |
| PPS1. The government has specific organizations or programs to help entrepreneurs develop new businesses. |
| PPS2. Governments at various levels provide diverse funding support and grant programs for entrepreneurs. |
| PPS3. The government has introduced various preferential policies to encourage entrepreneurial activities. |
| PPS4. The government prioritizes newly established businesses in public procurement or bidding projects. |
| PPS5. Even after a business failure, the government offers opportunities and support for re-entrepreneurship. |
| PPS6. I have access to relevant information about government policies and funding support for entrepreneurship. |
| PPS7. The local government where my university is located actively promotes and supports student entrepreneurship. |
| ***- Perceived entrepreneurial education*** |
| PEE1. I gained new experience and knowledge through the entrepreneurship course. |
| PEE2. The entrepreneurship course has helped me improve my entrepreneurial skills. |
| PEE3. The entrepreneurship course is related to real-world situations and is practically meaningful. |
| PEE4. The lecturers are experienced in teaching entrepreneurship courses. |
| PEE5. The lecturers use engaging and interactive teaching methods in entrepreneurship courses. |
| PEE6. Practical activities help me better understand entrepreneurship. |
| PEE7. My university fosters a strong entrepreneurial atmosphere. |
| PEE8. My university encourages and supports student participation in entrepreneurship. |
| PEE9. At university, I have had the opportunity to meet many people with entrepreneurial ideas. |
| PEE10. I believe university is the best place to receive systematic entrepreneurial education. |
| ***- Perceived access to finance*** |
| PAF1. If I were to start my own business, I know how to prepare a business plan. |
| PAF2. If I were to start my own business, I know how to use financial information to make decisions. |
| PAF3. I am familiar with some financing channels suitable for college student entrepreneurs (e.g., government funds, startup grants). |
| PAF4. I know where to find information related to entrepreneurial financing. |
| PAF5. I can access financing information through various channels (e.g., campus lectures, online, startup platforms). |
| PAF6. For me, obtaining startup capital for entrepreneurship is achievable. |
| PAF7. Compared to other entrepreneurial challenges, getting financial support is my biggest obstacle. |
| PAF8. Compared with traditional entrepreneurs, it is more difficult for college student. |
| **Role Models** |
| RM1. I consider this entrepreneurial role model as an example to follow. |
| RM2. I aspire to become like this entrepreneurial role model. |
| RM3. I am trying to develop the same qualities as this entrepreneurial role model. |
| RM4. I feel a strong emotional connection with this entrepreneurial role model. |
| **Entrepreneurial Intention** |
| EI1. I am considering creating my own business in the future. |
| EI2. I am prepared to do whatever it takes to become an entrepreneur. |
| EI3. I am trying to initiate and move forward with my own venture. |
| EI4. I am determined to launch my own business in the future. |
